# Words of Cheer

**Published:** 1870-06

**Authors:** 


### EDITORIAL DEPARTMENT.

*Words of Cheer.*—To give universal satisfaction, especially when laboring for the public good at a sacrifice of both time and means, has again and again been attempted, but only to end in failure, so diverse are the motives and opinions of the human race. It is satisfactory, however, to know that services, when they really possess merit, will always be appreciated by those whose opinions are of value, and who are above the jealousy and envy to which human nature is so prone.

To edit a professional journal is by no means an easy task, although it evidently appears so to some, who profess to know all concerning it without having had any experience whatever in the business. At one time we have suggestions as to how a dental periodical should be conducted so as to become popular, and at another time *hear* complaints that the work is not practical enough—in other words that it should have its pages filled with new methods for performing dental operations at a saving of both time, labor and expense, and pay no attention whatever to the collateral sciences, which, although they may improve the mind and increase the general knowledge so necessary in the practice of our profession, do not teach how the greatest amount of money may be obtained at the smallest expenditure of labor, time and means. It is encouraging, however, to know that such complaints arise only from those whose education has been so sadly neglected that they cannot now appreciate anything above their own standard, or whose nature is so warped by the rank growth of envy and jealousy, that the finer qualities are suppressed.

But when those who are competent to judge express favorable opinions and appreciate efforts exerted for the good of the profession, no heed need be paid to the class alluded to above, who may, perhaps, be more the objects of pity than of condemnation.

We have only space to refer at this time to a few of the many favorable opinions expressed concerning the *American Journal of Dental Science.* Our able and ever welcome *confrere*, *The Missouri Dental Journal*, at the close of a review of the contents of the February No. of this Journal, says: "It affords us great pleasure to be able to state that we consider *The American Journal of Dental Science* as one of the very best among our list of exchanges."

The Boston *Gynœcological Journal*, edited by Drs. Storer, Lewis and Bixby, so well known in the medical profession, says that the *American Journal of Dental Science*, "is edited with judgement and care, and deserves undoubtedly a generous support."

These are "words of cheer" coming from those whose opinions we value.

